# Babelomics 5.0: functional interpretation for new generations of genomic data

**DOI:** 10.1093/nar/gkv384

**Published:** 2015-04-20

**Authors:** Roberto Alonso, Francisco Salavert, Francisco Garcia-Garcia, Jose Carbonell-Caballero, Marta Bleda, Luz Garcia-Alonso, Alba Sanchis-Juan, Daniel Perez-Gil, Pablo Marin-Garcia, Ruben Sanchez, Cankut Cubuk, Marta R. Hidalgo, Alicia Amadoz, Rosa D. Hernansaiz-Ballesteros, Alejandro Alemán, Joaquin Tarraga, David Montaner, Ignacio Medina, Joaquin Dopazo

**Affiliations:** 1 Computational Genomics Department, Centro de Investigación Príncipe Felipe (CIPF), Valencia, 46012, Spain; 2 Computational Genomics Chair, Bull-CIPF, Valencia, 46012, Spain; 3 Bioinformatics of Rare Diseases (BIER), CIBER de Enfermedades Raras (CIBERER), Valencia, 46012, Spain; 4 Department of Medicine, University of Cambridge, School of Clinical Medicine, Addenbrooke's Hospital, Hills Road, Cambridge CB2 0QQ, UK; 5 Fundación Investigación Clínico de Valencia-INCLIVA, Valencia, 46010, Spain; 6 Functional Genomics Node, (INB) at CIPF, Valencia, 46012, Spain; 7 HPC Services, University of Cambridge, Cambridge, CB3 0RB UK

## Abstract

Babelomics has been running for more than one decade offering a user-friendly interface for the functional analysis of gene expression and genomic data. Here we present its fifth release, which includes support for Next Generation Sequencing data including gene expression (RNA-seq), exome or genome resequencing. Babelomics has simplified its interface, being now more intuitive. Improved visualization options, such as a genome viewer as well as an interactive network viewer, have been implemented. New technical enhancements at both, client and server sides, makes the user experience faster and more dynamic. Babelomics offers user-friendly access to a full range of methods that cover: (i) primary data analysis, (ii) a variety of tests for different experimental designs and (iii) different enrichment and network analysis algorithms for the interpretation of the results of such tests in the proper functional context. In addition to the public server, local copies of Babelomics can be downloaded and installed. Babelomics is freely available at: http://www.babelomics.org.

## INTRODUCTION

Babelomics is an integrative web-based platform for the functional analysis of transcriptomic and genomic data. Named after the tale ‘The Babel library’ (1), a masterpiece by the famous Argentinean writer Jorge Luís Borges that describes an infinite library containing all the possible books, Babelomics has been running for more than 10 years, becoming rapidly a classic in the field of functional analysis. Its first version, published in 2005 (2), consisted of a collection of methods for functional enrichment analysis (3,4) based on different biologically relevant terms (GO; Gene Ontology, KEGG, etc.). Since then, Babelomics has released new versions that incorporated transcriptomics primary data analysis methods from the GEPAS (5–9) (a web tool discontinued by the end of 2012). The functionality of these new versions was complemented with more functional analyses, such as network analysis (10,11), or text-mining (12). Also, the possible data types were expanded to single nucleotide polymorphisms (SNPs) and thus Genome Wide Association Analysis (GWAS) could be carried out in Babelomics (13–15). In terms of software, Babelomics has evolved by adopting increasingly efficient web technologies. Thus, from the plain HTML of the initial versions (2,14), Babelomics was re-engineered to use SOAP web services and Web 2.0 technology features, such as AJAX in the 2008 release (13). Later, in the 2010 release (15), the backend was rewritten in Java while an extensive use of JavaScript at the client side was made. The continuous adoption of new technologies, such as the HTML5 standard and RESTful web services, has enabled the design of successive interfaces that allow asynchronous use, as well as the management of projects, jobs and user accounts.

Babelomics provides easy-to-use solutions for the most common scenarios of genomic and transcriptomics data analysis, offering the possibility of exploring the effects of alteration in gene expression levels or changes in genes sequences within a functional context (GO classes, interactome, etc.) Typical Babelomics users are laboratory researchers with no programming skills but have a hypothesis they want to test using their data. The use of Babelomics accelerates the discovery process in the laboratories and reduces the routine work load in the bioinformatics and biostatistics facilities thus contributing to the optimization of the whole research ‘ecosystem’. Babelomics has also been extensively used in courses. Currently, an average of more than 200 experiments per day are analysed in Babelomics, (http://bioinfo.cipf.es/webstats/babelomics/awstats.babelomics.bioinfo.cipf.es.html), distributed across many different countries (http://bioinfo.cipf.es/toolsusage). The four Babelomics publications have received a total of 522 citations (recorded by 21 January 2015, according to Thomson Reuters’ Web of Science, https://webofknowledge.com).

As a response to the changes in the technologies this new version of Babelomics includes the possibility of analysing genomic and transcriptomics data from new generation sequencing (NGS) experiments. Thus, in addition to conventional microarray data, RNA-seq data and massive resequencing data can now be uploaded and analysed. A detailed analysis of use during the last four years has been used for discontinuing a number of options that have been scarcely used. This has resulted in a simplified, more intuitive and more sustainable interface for Babelomics. New advanced options for visualization have been implemented. These include a network viewer for the representation of the results of network analysis, which allows users to customize the results and to produce high quality figures for papers. Also a genome browser, Genome Maps (16), which allows visualizing SNPs or variants in their genomic context, was included.

On the other hand, huge datasets from high-throughput technologies bring about new challenges for data analysis and visualization. To keep pace with this data revolution Babelomics web interface has been redesigned and rewritten using new web technologies. Also a radical restructuring has been done at the server side to speed it up and make the analysis faster and more efficient.

We have reduced the dependence on many external databases, which made the update of the relevant information difficult in previous versions. Babelomics now uses CellBase (17) as unique source of information. CellBase currently resides at the European Bioinformatics Institute (EBI) and it is updated on regular basis.

Summarizing, Babelomics 5.0 includes support for new genomic data from NGS experiments and new analysis options. From a technological point of view it includes new visual and web technologies that provide a more robust, fast and interactive interface.

## BABELOMICS STRUCTURE

Babelomics is structured in four conceptually different parts represented in the main menu: *Processing, Expression, Genomics, Cancer* and *Functional*. In addition, the menu bar contains the data entry point, *Upload*, the *Jobs* manager and the question mark icon that contains the tutorial, contact and credits.

Babelomics relies on a series of powerful resources developed by us in the last years. Now, CellBase (17) provides all the functional information required in the different steps of analysis via highly optimized RESTful web services (see https://github.com/opencb/cellbase/wiki). Innovative visualization interfaces have been implemented in Babelomics. An interactive and highly efficient genome browser, Genome Maps (16), allows representing variants (or any other genomic feature) in its genomic context. Network analysis results are now visualized in a new interactive visual framework, CellMaps (https://github.com/opencb/cell-maps/wik), which can produce high-quality figures customized by the user.

### Data upload and WorkSpace

Babelomics can be used either in anonymous mode or as a registered user. In anonymous mode, all the uploaded data and the results obtained (but not saved in the user's terminal) are lost at the end of the session. In registered user mode the options are the same, the only difference is that registered users can maintain the data and the results in the Babelomics workspace with a limit of 10 GB (that can be changed in local installations of Babelomics). Registration is free. The workspace structure has improved with respect to previous versions and has the familiar appearance and functionality of the typical file system. The upload option of the main menu brings about the WorkSpace, where data files can be uploaded. Data files can also be uploaded from within any analysis option of the menu. Different analysis options in the menu can have specific format requirements. As a general rule, data consist on raw sequencing (VCF or counts) or microarray (.CEL, etc.) files in the first steps (Preprocessing). In subsequent analysis steps, the files are taken properly formatted from the previous steps. Nevertheless, files can be preprocessed and analysed with other tools and uploaded at later analysis steps providing they are properly formatted.

### Data processing

Microarray normalization contains the same options that Babelomics 4 offered, which includes support for Affymetrix normalization and both, one-channel and two-channel normalization for Agilent and Genepix.

Regarding RNA-seq normalization, we have included an automatic decision rule to suggest the most suitable normalization method, depending on the potential biases detected in the data. The main factors that can originate biases are: library depth (irrelevant for samples from the same library), gene length and extreme differences in mRNA abundances. Thus, if a clear mRNA composition bias is detected, *Trimmed Means of M-values* (TMM) normalization (18) is recommended, otherwise *Reads Per Kilobase per Million* (RPKM) method (19) is preferable. Noiseq (20) is used to carry out bias detection and the result produces the pre-selection of the normalization method (that obviously can be changed by the user).

In addition, there is an improved option for attribute edition that allows editing variable and label names in the data. Another option allows several transformations over the data matrices (including normalization, logarithm transformation, missing value imputation, etc.)

### Expression data analysis

Typical expression data analyses include unsupervised analysis (clustering) and supervised analysis (differential expression or classifiers). These microarray data analyses are the same as in Babelomics 4. Actually, previous Babelomics versions implemented new clustering methods especially devised for clustering large datasets, such as the SOTA (21) and pioneered the implementation of classifiers in web tools (22).

The supervised analyses can be carried out with RNA-seq data as well. The method used for differential expression in RNA-seq data is different from the tests used for microarrays, given the different statistical distributions followed by both data types. RNA-seq counts are transformed with the *Voom* method (23) that allows subsequent linear analysis using *limma* (24). As in the case of microarray differential expression tests, different multiple-test correction methods are available.

### Genomic data analysis

This module aims to give support to simple case/control or transmission disequilibrium test (TDT) experiments in Genome Wide Association Studies (GWAS) in Babelomics 4. The popular PLINK software (25) is used to carry out the tests. The results include a Manhattan plot, a list of SNPs and, below, a new graphical interface, provided by an embedded version of the genome viewer Genome Maps (16), that allows exploring significant SNPs or variants in its genomics context. In this new version we have also included one extensively used burden test for the analysis of sequence data, the Combined Multivariate and Collapsing (CMC) method (26). In particular, the regions defined here are genes (given that the most common NGS data is still produced by exome sequencing), that can be further analysed in the *Functional* data analysis module.

### Cancer

In the last years, cancer genomics has experienced a data generation revolution. The completion of two large international initiatives, the Cancer Genome Atlas (27) (http://cancergenome.nih.gov/) and the International Cancer Genome Consortium (28) (https://icgc.org/) has made available a huge amount of genomic data. Thus, whole exome and genome sequencing of cancer samples is becoming mainstream. Here we have integrated two popular tools specifically devised for the analysis of cancer genomic sequences. One of them is Oncodrive-FM (29), which computes a metric of functional impact using three well-known methods, SIFT (30), PolyPhen (31) and MutationAssessor (32). This metric is used to detect potential cancer driver genes by studying how the functional impact of variants found across several tumour samples deviates from a null distribution. The other tool, OncodriveCLUST (33), aims to identify genes undergoing mutations that tend to be clustered instead of being evenly distributed within them. This method is designed to exploit the observation that mutations in cancer genes, especially oncogenes, often cluster in particular positions of the protein. Both methods allow detecting genes of potential relevance in cancer, within variant files (in VCF format), which can be further analysed in its functional context in the *Functional* data analysis module.

### Functional data analysis

The differential aspect of Babelomics with respect to other similar tools is that any result in terms of (often not very informative) lists of genes with *P*-values obtained in any of the analysis modules above described (*Expression, Genomics* and *Cancer*) can internally be submitted to the *Functional* data analysis module where they can be interpreted within different functional contexts. A simple way to assess the possible functional roles played by a list of genes consists on studying the distribution of functional annotations associated to these genes. GO (34) is the most extensively used source of functional annotations for genes. *Single Enrichment* methods study over-representations on any of these GO terms in the resulting lists obtained in previous analysis modules (e.g. differentially expressed genes, genes containing SNPs or variants associated to the disease, etc.) The popular *FatiGO* algorithm (3), already present in early Babelomics versions, implements the single enrichment method also in this release. Similarly, the *Gene Set Enrichment* algorithm, now common to both, *Expression* (4) and *Genomic* (35) data, is included in the new Babelomics as well. In a similar way, lists of genes can be interpreted in the context of the interactome. Thus, *Network Enrichment* analysis finds the largest significant network that can be formed with the genes contained in a list (10). On the other hand, *Gene set network enrichment* analysis uses an ordered list of genes to find the network significantly associated to the highest values of the list (typically the lowest *P*-values of a test from any of the analysis modules) (11). Gene Set Enrichment methods are known to be more sensitive than Single Enrichment methods (36,37). At present, eight species representative of the main organism models are supported: *Homo sapiens, Mus musculus, Rattus norvegicus, Danio rerio, Drosophila melanogaster, Caenorhabditis elegans, Arabidopsis thaliana* and *Saccharomyces cerevisiae*. More species will be supported in the future, although users can upload their own annotations for other species.

A new visual framework, CellMaps (https://github.com/opencb/cell-maps/wik), is provided in this new Babelomics version. This framework provides a smart and interactive representation of the resulting networks that allows users to produce customized high-quality figures, ready for the publisher.

## BEHIND THE SCENE: THE BABELOMICS SERVER AND THE CLIENT

On the server side, some algorithms have been rewritten in C/C++ in order to take now advantage of the modern multi-core CPU capabilities of the hardware in which Babelomics runs. Also, the results of some analysis have been indexed to speed up queries from the web site. On-fly-time GZIP conversion has also improved data transfer for both downloads and uploads. Web services have been re-implemented using RESTful web services, which are lighter and have a low latency. CellBase (17), the database where Babelomics extracts information from, has been redesigned and transformed into a NoSQL database. This provides a high-performance and scalable solution to cope with increasingly complex and bigger queries. CellBase was originally born as an internal relational database to store biological data for Babelomics and was later released as independent project (17). During the last year, Babelomics and EBI developers have worked together to migrate CellBase to MongoDB, one of the most successful NoSQL databases. MongoDB is a high-performance and scalable document-oriented database, that makes easy to add big and complex data and provides a rich API to execute complex queries.

The client has been completely rewritten using new technologies and standards. Babelomics is now entirely implemented in HTML5 and makes use of other technologies such as Scalable Vector Graphics (SVG) for visualization or IndexedDB for caching information and minimizing the queries to the server. As part of this effort Babelomics has significantly contributed to develop Genome Maps and CellMaps, which have been integrated to offer high quality visualization capabilities to Babelomics users. Also, new Web Components standard is being used now. This allows the community to reuse most of the visual components developed. Due to the intensive use of cutting-edge web technologies only modern web browsers are fully supported, these include Chrome 36+, Firefox 36+, Safari 8+ and Opera 24+.

Apart from using the public version of Babelomics, the code is open and freely available for local installation. The code can be found in GitHub: https://github.com/babelomics/babelomics.

## FUTURE PROSPECTS

The roadmap of Babelomics for the near future includes: more conventional tests for relatively common experimental designs not fully covered in this version, more context for the functional analysis and full integrative analysis of data and information. An obvious step ahead in functional analysis will be the inclusion of pathway analysis. We are working on the integration of models of signalling pathways (38,39) in future versions of Babelomics. Another important aspect is the regulatory information. Methods for inferring the regulatory circuit behind a transcriptomics experiment already developed (40) will also be included. Additionally, integrative analysis of complex experimental designs, in which genomic and transcriptomics data are simultaneously obtained, will be included. Finally, support for more species will soon be added. Since the functional information relies on CellBase (17), the inclusion of more species is straightforward.

## CONCLUSIONS

Babelomics has evolved again and in this fifth version it incorporates next generation sequencing data, new analysis options and new technologies at both, server and client sides. It offers a user-friendly environment that provides a full range of solutions which include primary data analysis, followed by a bunch of test for different experimental designs and data types and completed with the possibility of testing the biological relevance of the results obtained within a functional context. One of the most distinctive features of Babelomics, the functional analysis of genomic data, is nowadays a critical aspect in data analysis. Given the multigenic nature of most traits, these can only be explained as the result of complex interactions between genes (41), a notion proposed more than a decade ago in the context of systems biology (42). Consequently, most diseases are better understood as failures of functional modules caused by different combinations of mutated genes rather than by unique mutation(s) in one single gene (43). The use of a Systems Biology perspective in the analysis of genomic data is leading to new approaches in biomedical research including diagnostics (44), drug discovery (45), as well as pharmacology and toxicology (46). The availability of a tool that offers user-friendly solutions to most of the conventional genomic analysis problems, complemented with an advanced functional analysis, within an environment that allows storing data and results, explains the success of Babelomics for more than a decade.
